# Identification of osteoporosis using ensemble deep learning model with panoramic radiographs and clinical covariates

**DOI:** 10.1038/s41598-022-10150-x

**Published:** 2022-04-12

**Authors:** Shintaro Sukegawa, Ai Fujimura, Akira Taguchi, Norio Yamamoto, Akira Kitamura, Ryosuke Goto, Keisuke Nakano, Kiyofumi Takabatake, Hotaka Kawai, Hitoshi Nagatsuka, Yoshihiko Furuki

**Affiliations:** 1 Department of Oral and Maxillofacial Surgery, Kagawa Prefectural Central Hospital, 1-2-1, Asahi-machi, Takamatsu, Kagawa 760-8557 Japan; 2 Department of Oral Pathology and Medicine, Graduate School of Medicine, Dentistry and Pharmaceutical Sciences, Okayama University, Okayama, 700-8558 Japan; 3 Department of Oral and Maxillofacial Radiology, School of Dentistry, Matsumoto Dental University, 1780 Hirooka Gobara, Shiojiri, Nagano 399-0781 Japan; 4 Department of Epidemiology, Graduate School of Medicine, Dentistry and Pharmaceutical Sciences, Okayama University, Okayama, 700-8558 Japan; 5 Search Space Inc., Tokyo, 151-0072 Japan

**Keywords:** Calcium and phosphate metabolic disorders, Panoramic radiography

## Abstract

Osteoporosis is becoming a global health issue due to increased life expectancy. However, it is difficult to detect in its early stages owing to a lack of discernible symptoms. Hence, screening for osteoporosis with widely used dental panoramic radiographs would be very cost-effective and useful. In this study, we investigate the use of deep learning to classify osteoporosis from dental panoramic radiographs. In addition, the effect of adding clinical covariate data to the radiographic images on the identification performance was assessed. For objective labeling, a dataset containing 778 images was collected from patients who underwent both skeletal-bone-mineral density measurement and dental panoramic radiography at a single general hospital between 2014 and 2020. Osteoporosis was assessed from the dental panoramic radiographs using convolutional neural network (CNN) models, including EfficientNet-b0, -b3, and -b7 and ResNet-18, -50, and -152. An ensemble model was also constructed with clinical covariates added to each CNN. The ensemble model exhibited improved performance on all metrics for all CNNs, especially accuracy and AUC. The results show that deep learning using CNN can accurately classify osteoporosis from dental panoramic radiographs. Furthermore, it was shown that the accuracy can be improved using an ensemble model with patient covariates.

## Introduction

Osteoporosis is defined by the loss of bone mass and the deterioration of the microarchitecture of bone tissue^1^. It is a common and potentially metabolic bone disease characterized by susceptibility to fracture. Fractures of the spine, hips, and wrists caused by osteoporosis significantly impair the quality of life of patients. In addition, in severe cases, it can lead to disorders that increase the risk of mortality^2^. With the rapid aging of the population caused by the increase in life expectancy in recent years, millions of people are affected annually worldwide, and osteoporosis is becoming a global public health problem. However, osteoporosis initially develops without any symptoms and can go undetected in its early stages^3^.

Dual-energy X-ray absorptiometry (DXA) is an effective means of identifying bone mineral density (BMD) and is the standard test for diagnosing osteoporosis^4^. Despite being standard inspection methods, DXA scans are relatively expensive^5^, which makes them unsuitable for general screening. Dental panoramic radiographs are frequently taken during regular dental examinations or before certain dental procedures. Therefore, it would be of great medical and economic value if dentists could use dental panoramic radiographs to screen patients for osteoporosis. This approach is also clinically useful in that dentists can refer patients with suspected osteoporosis to specialists. Several researchers have analyzed dental panoramic radiographs to provide initial diagnoses of osteoporosis^6–16^.

The detection of osteoporosis using panoramic radiographs has been investigated in relation to several concentrations and linear measurements, such as the mandibular cortical width (MCW), mandibular cortex index (MCI), mental index, and panoramic mandibular index^6–16^ In addition, the diagnosis of osteoporosis using a support vector machine has been reported^15^. However, these diagnostic imaging methods have not been commonly used because they require complicated preprocessing, image normalization, and complicated and specialized measurements for diagnosis. In contrast, the diagnosis of osteoporosis by deep learning using a convolutional neural network (CNN) that does not require complicated pretreatment has also been reported. One study that used deep learning focusing on the mandibular cortical bone produced a high diagnostic accuracy of 84.0%, and an area under the curve (AUC) of the receiver operating characteristic (ROC) curve of 0.858^17^. It has been suggested that deep learning using X-ray images can be useful for diagnosing osteoporosis.

The conventional methods of classifying osteoporosis by extracting each feature from panoramic images are extremely useful. However, osteoporosis is associated with systemic patient factors^18^. We hypothesized that the diagnostic accuracy using deep learning and X-ray images would be improved by constructing a CNN in which patient factors are added.

The purpose of this study was to construct an osteoporosis classifier from dental panoramic radiographs. In addition, we developed an osteoporosis classifier based on an ensemble model in which the clinical covariates of patients were added to dental panoramic radiographs to statistically clarify the effect of classification accuracy on the addition of clinical covariates.

## Results

### Prediction performance

#### Comparison between image-only model and ensemble model

Table 1 shows the performance metrics, *P*-values, and effect sizes for ResNet-18, -50, and -152. All performance metrics were elevated using the ensemble model. Both the image-only model and the ensemble model showed higher performance in the order of ResNet-18, -50, and -152. There is a strongly statistically significant difference between the two groups, especially in terms of accuracy and AUC. In the effect size evaluation, the AUC had the highest effect in all ResNet models, categorized as very large.

**Table 1:** Comparison of performance metrics in ResNet.

	Accuracy	AUC score	Precision	Recall	Specificity	F1 score
	SD	SD	SD	SD	SD	SD
	95%CI	95%CI	95%CI	95%CI	95%CI	95%CI
**ResNet-18**						
Image-only model	0.809	0.874	0.745	0.605	0.898	0.646
	0.012	0.010	0.033	0.065	0.021	0.045
	0.804–0.813	0.870–0.878	0.733–0.757	0.581–0.630	0.890–0.906	0.629–0.662
Ensemble model	0.824	0.893	0.768	0.630	0.909	0.676
	0.012	0.011	0.024	0.050	0.016	0.033
	0.819–0.828	0.889–0.898	0.759–0.777	0.611–0.649	0.903–0.915	0.664–0.688
*P* value	< 0.0001	< 0.0001	0.003	0.103	0.029	0.004
Effect size	**1.227**	**1.849**	**0.803**	0.422	**0.570**	0.761
**ResNet-50**						
Image-only model	0.826	0.890	0.752	0.661	0.899	0.691
	0.010	0.011	0.029	0.049	0.017	0.029
	0.822–0.829	0.886–0.894	0.741–0.763	0.643–0.679	0.892–0.905	0.680–0.702
Ensemble model	0.837	0.905	0.773	0.684	***0.906***	0.714
	0.011	0.009	0.028	0.041	0.018	0.023
	0.833–0.841	0.901–0.908	0.762–0.783	0.668–0.699	0.899–0.912	0.706–0.723
*P* value	< 0.0001	< 0.0001	0.006	0.056	0.130	0.001
Effect size	1.118	1.393	0.725	0.498	0.392	**0.887**
**ResNet-152**						
Image-only model	0.830	0.895	0.764	0.665	0.903	0.699
	0.011	0.011	0.028	0.046	0.018	0.030
	0.825–0.834	0.891–0.899	0.754–0.774	0.648–0.682	0.896–0.909	0.687–0.710
Ensemble model	**0.840**	**0.911**	**0.774**	**0.695**	**0.906**	**0.720**
	0.009	0.008	0.028	0.045	0.020	0.025
	0.837–0.844	0.908–0.914	0.764–0.785	0.678–0.712	0.898–0.913	0.711–0.729
*P* value	< 0.0001	< 0.0001	0.169	0.013	0.552	0.004
Effect size	1.056	1.625	0.355	**0.652**	0.153	0.764

Bold showed the highest effect size in each performance metric and bold italics showed the highest score in each performance metric.

Table 2 shows the performance metrics, *P*-values, and effect sizes for EfficientNet-b0, -b3, and -b7. As with ResNet, all performance metrics are increased by the ensemble model. Both the image-only model and ensemble model show higher performance in the order of EfficientNet-b0, -b3, and -b7. The two-group comparison also showed strong statistically significant differences in accuracy and AUC, and the effect sizes were all very large. Among all CNN models, EfficientNet-b7 produced the highest accuracy, AUC, and F1 score. The effect sizes tended to be higher for models with fewer parameters in both ResNet and EfficientNet. It has been shown that the ensemble model is more effective in case of small number of parameters. (Bold in Tables 1 and 2 shows the highest effect size in each performance metric. Bold italics shows the highest score in each performance metric.) Interestingly, an ensemble model with additional clinical variables in multiple CNN models, regardless of the number of parameters, contributed to improved performance. Figure S1 shows the ROC curves corresponding to ResNet and EfficientNet.

**Table 2:** Comparison of performance metrics in EfficientNet.

	Accuracy	AUC score	Precision	Recall	Specificity	F1 score
	SD	SD	SD	SD	SD	SD
	95%CI	95%CI	95%CI	95%CI	95%CI	95%CI
**EfficientNet-b0**						
Image-only model	0.792	0.844	0.695	0.590	0.882	0.627
	0.015	0.027	0.043	0.067	0.022	0.069
	0.786–0.797	0.834–0.854	0.679–0.711	0.564–0.615	0.874–0.890	0.602–0.653
Ensemble model	0.811	0.882	0.726	0.634	0.890	0.661
	0.015	0.015	0.034	0.038	0.018	0.032
	0.805–0.816	0.877–0.888	0.714–0.739	0.620–0.648	0.884–0.897	0.649–0.673
*P* value	< 0.0001	< 0.0001	0.003	0.003	0.114	0.020
Effect size	**1.263**	1.738	**0.804**	0.803	**0.409**	0.612
**EfficientNet-b3**						
Image-only model	0.807	0.867	0.711	0.635	0.883	0.655
	0.016	0.018	0.035	0.058	0.020	0.045
	0.801–0.813	0.860–0.874	0.698–0.724	0.613–0.657	0.875–0.891	0.638–0.672
Ensemble model	0.824	0.899	0.733	0.680	0.887	0.692
	0.013	0.014	0.026	0.051	0.016	0.036
	0.819–0.829	0.894–0.904	0.723–0.742	0.661–0.699	0.881–0.893	0.679–0.705
*P* value	< 0.0001	< 0.0001	0.008	0.002	0.395	0.001
Effect size	1.110	**1.962**	0.698	**0.815**	0.218	**0.907**
**EfficientNet-b7**						
Image-only model	0.832	0.900	0.743	0.716	0.884	0.716
	0.011	0.011	0.025	0.049	0.018	0.029
	0.828–0.836	0.896–0.904	0.734–0.752	0.698–0.734	0.877–0.890	0.705–0.726
Ensemble model	**0.845**	**0.921**	**0.752**	**0.749**	**0.888**	**0.740**
	0.013	0.012	0.027	0.055	0.021	0.032
	0.841–0.850	0.917–0.925	0.742–0.763	0.729–0.770	0.880–0.895	0.728–0.752
*P* value	< 0.0001	< 0.0001	0.172	0.015	0.449	0.003
Effect size	1.101	1.780	0.352	0.636	0.194	0.790

Bold showed the highest effect size in each performance metric and bold italics showed the highest score in each performance metric.

### Visualization of model identification

**Figure 1:**
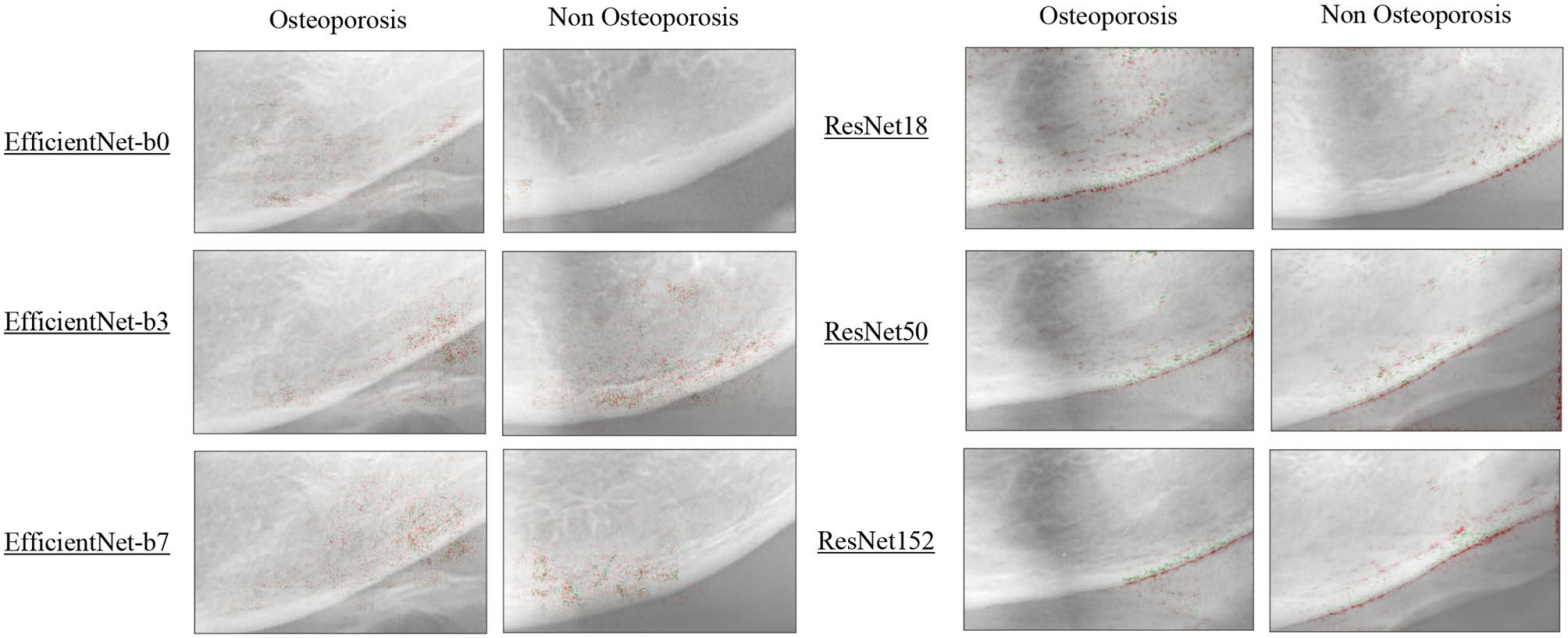
Visualization of characteristic regions of radiographs of osteoporosis and non-osteoporosis patient images using ResNet and EfficientNet.

Figure 1 shows the focused visualization area obtained by guided Grad-CAM. We selected the ensemble analysis using EfficientNet-b0, -b3, and -b7 and ResNet-18, -50, and -152. Both EfficientNet and ResNet commonly focused on the cortical bone region of the mandibular lower border as a feature region. EfficientNet determined that this area was a characteristic region in non-osteoporosis images. In contrast, in the osteoporosis images, the area above the cortical bone was judged to be a characteristic region in addition to the cortical bone region of the mandibular lower border. ResNet characterized the cortical bone at the lower edge of the mandible more strongly. In osteoporosis images, ResNet-50 and -152 paid particular attention to the mandibular lower border cortical bone. ResNet did not consider the area above the mandibular cortical bone as a characteristic region, whereas EfficientNet did. In the non-osteoporosis images, the cortical bone in the entire mandibular lower border was judged to constitute a characteristic region. In both EfficientNet and ResNet, the larger the number of parameters, the smaller the variation in the area that captured the image features.

## Discussion

This study demonstrates that CNNs can diagnose osteoporosis from dental panoramic radiographs with high levels of accuracy. Moreover, including patient variables involved in routine clinical settings improved the performance metrics of all predictions compared to using the image-only model. In particular, the ensemble model was more effective for the CNN model with fewer parameters.

There was no significant difference in diagnostic accuracy for our images compared to previous reports of osteoporosis classification by deep learning using dental panoramic radiographs^17^. The advantage of the method applied in this work is that we created a model with clinical patient covariates added to improve the accuracy of deep learning using images. This article is the first to report on the identification of osteoporosis using an ensemble model from dental panoramic radiographs. The addition of patient covariates provided additional information regarding important osteoporosis classifications and improved all performance metrics over the image-only model. In particular, the accuracy and AUC were statistically significantly improved by the sample model.

It is presumed that the diagnostic accuracy was improved because advanced inference was enabled by deep learning that simultaneously considers important information related to clinical covariates that cannot be extracted from dental panoramic X-ray images alone.

In this study, we used ResNet and EfficientNet CNNs. In general, CNNs have a deep hierarchical structure to improve accuracy. ResNet-152 and EfficientNet-B7 showed the highest accuracy among the ResNet and EfficientNet approaches, respectively. This finding is consistent with the results of previous studies^19,20^. In addition, performance improvements were obtained in all cases from the CNNs with few parameters compared to the CNNs with numerous parameters. Although the increase in the effect size of the ensemble became smaller as the number of parameters increased, it was suggested that the ensemble model is effective for achieving higher performance.

In our study, using patient clinical covariate data structured with images was more efficient in classifying osteoporosis by deep learning than using images alone. Only a few scholars have employed images using deep learning and ensemble models with clinical covariates^21,22^ Clinical data that reflect the general condition of the patient are important factors in the diagnosis of osteoporosis^23^. However, unfortunately, it is difficult to collect highly specialized clinical information such as accurate histories of fractures and time of menopause from first-time patients at dental clinics. Our study envisaged a more accurate screening method for dentists involving panoramic radiographs. We created an ensemble model with relatively high osteoporosis classification accuracy using age, gender, and BMI, which are easily collectable and clinically important data, as clinical covariates.

In this study, we used guided Grad-CAM technology to visualize feature regions in deep learning. The visualization of the feature area was different between ResNet and EfficientNet, and this result was extremely interesting. ResNet focused on the cortical bone in the mandibular lower border. In contrast, EfficientNet focused on the area above the cortical bone in addition to the cortical bone in the mandibular lower border. In previous studies, the MCW and MCI were used as indicators in osteoporosis screening^8,9,11,14^. MCI is a screening method that focuses on structural changes in the cortical bone due to bone resorption^24^. It is presumed that ResNet mainly focused on the MCW, whereas EfficientNet regarded both the MCW and MCI as characteristic areas. The MCW may not have shown the ability to detect osteoporosis^25^, and the MCI was not reproducible, which were drawbacks of these measurement methods^14^. The MCW is characterized by higher specificity than sensitivity^26^. It was speculated that ResNet showed higher specificity mainly due to the MCI and derived from its characteristic region. The high classification performance of EfficientNet may be due to its focus on each of the two measurement methods.

The advantage of this study over previous works is the statistical assessment of the additional effects of patient factors on the identification of osteoporosis from panoramic radiographs using deep learning. To the best of our knowledge, this study is the first to adopt this approach. In addition, the effect sizes calculated in this study will facilitate sample size estimation in future works.

This study has three notable limitations. Although we utilized more cases than previous research^17^, it was difficult to collect sufficient image data from a single general hospital. CNNs with a small amount of data can lead to overfitting and reduced generalization. We organized the data to avoid overfitting and used cross-validation and early-stopping learning methods. In general, models trained by deep learning from large image datasets are effective for image classification. By increasing the amount of data through multi-center collaborative research, the accuracy and generalization of CNN classification diagnosis can be improved. The second limitation is the type of CNN adopted for validation. In this study, EfficientNet and ResNet were examined at various depths. If a CNN with fewer parameters could achieve higher performance, it would be more widely applicable as the calculation cost would thus decrease. The identification of various CNNs suitable for image quality and patient covariate ensembles remains as an important task for future research. In this study, the images were manually cropped to include the mandibular inferior margin in the center of the mandibular body as a preoperative preparation to classify osteoporosis. In future, the construction of a network that can screen for osteoporosis from dental panoramic radiographs by automatically detecting the ROI from untrimmed dental panoramic radiographs is required. Specifically, it is expected to be used in combination with object detection methods such as region-based CNN, single-shot multi-box detector^27^. Muramatsu et al. reported on the automatic detection of MCI^28^, which could be applied to the setting of ROIs. Furthermore, it is ideal to ensemble patient covariates automatically by linking them with electronic medical record information. It is desired to verify the effectiveness of the ensemble model using a new deep learning model that is lighter and more accurate. Another limitation is the comparison of the results of deep learning. In this study, we examined the effectiveness of the ensemble model using CNN. Although the ensemble model has been shown to contribute to improved accuracy, it remains unclear if it is superior to clinicians. In future, it will be necessary to compare this model with clinicians and verify whether the accuracy of clinicians’ identification changes by allowing them to refer to the areas indicated by deep learning techniques. These verifications will contribute to the development of deep learning.

## Conclusions

Using deep learning with the CNN model demonstrated that osteoporosis can be classified with relatively higher accuracy from dental panoramic radiographs. In addition, an ensemble model that included patient covariates demonstrated more accurate classification of osteoporosis. The ensemble model contributed to the performance improvement in all the CNN models and was more effective for the CNN model with fewer parameters. The EfficientNet-B7 and ResNet-152 ensemble models were also classified with highest accuracy. These results are expected to play an important role in the screening of osteoporosis in the clinical dental environment.

## Materials and methods

### Study design

The aim of this study was to classify osteoporosis and non-osteoporosis using a dataset segmented from panoramic radiographs and several different CNNs. Supervised learning was employed as a deep learning method. We statistically investigated the effect of adding covariates extracted from clinical records on the accuracy of the osteoporosis identification.

### Data acquisition

We retrospectively used clinical and radiographic data from March 2014 to September 2020. This study protocol was approved by the institutional review boards of the respective institutions hosting this work (i.e., the review boards of Kagawa Prefectural Central Hospital, approval number 994), following Ethical guidelines for clinical research and in accordance with the ethical principles that have their origins in the Declaration of Helsinki and its subsequent amendments. Informed consent from individual patients for this retrospective study was waived at the discretion of the institutional review committee (Kagawa Prefectural Central Hospital Ethics Committee) because protected health information was not used. The study included 902 consecutive images from enrolled patients who underwent panoramic radiography within the first year of receiving DXA at our hospital.

Osteoporosis was diagnosed by the DXA method using the hip or spine. The parameters investigated included the automatically generated BMD (g/cm^3^) and T-score. Osteoporosis was diagnosed when the T-score of the BMD was less than − 2.5 and non-osteoporosis when the T-score was − 2.5 or more, according to the diagnostic criteria of the World Health Organization^29^. When DXA was performed at both the hip and spine sites, the result with the lower T-score was used for diagnosis.

The following panoramic radiographs were excluded from this study: 119 images of patients taking antiresorptive agents such as bisphosphonates or anti-RANKL antibodies, 3 images of foreign substances such as plates and gastric tubes, 1 image of a mandibular fracture, and 1 image with poor panoramic radiography. Further analysis was conducted on the remaining 778 images.

### Data preprocessing

**Figure 2:**
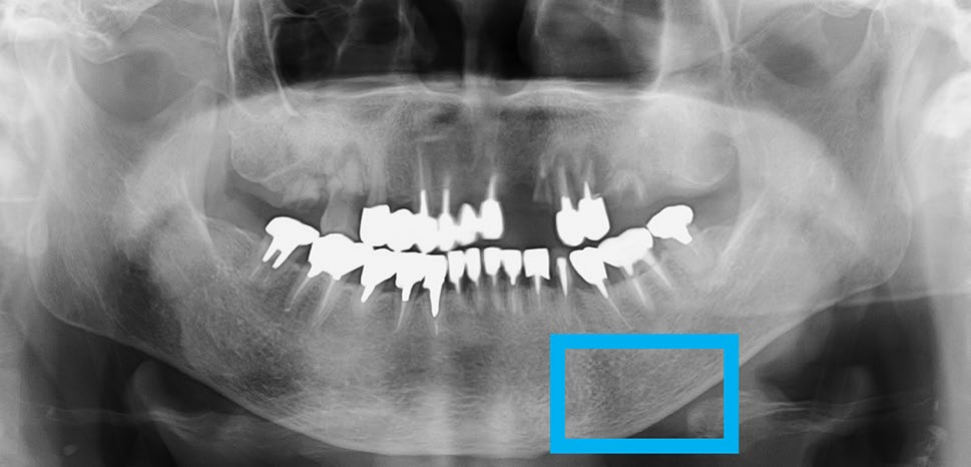
Dental panoramic radiographs before deep learning analysis, showing cropped ROI.

Dental panoramic radiographs of each patient were utilized to acquire images using an AZ3000CMR (ASAHI ROENTGEN IND. Co., Ltd., Kyoto, Japan). All data images were output in .tiff format (2964 × 1464 pixels) from the Kagawa Prefectural Central Hospital PACS system (HOPE DrABLE-GX, FUJITSU Co., Tokyo, Japan). We isolated the cortical bone at the lower edge of the mandible in the images. Two maxillofacial surgeons manually placed and cropped regions of interest (ROIs) on the dental panoramic radiograph images using Photoshop Elements (Adobe Systems, Inc., San Jose, CA, USA). The ROI was set according to previous studies of deep learning that identified the ROI in osteoporosis by panoramic radiography. A previous study identified the middle area of the mandibular lower border as the ROI^17^. To ensure reproducibility, the mental foramen was used as the reference point at the mid-point of the mandible. The ROI was created to be 250 × 400 pixels in size just below the reference point to include the lower edge of the mandible. All analyses in this study were performed on the left side, as shown in Fig. 2. The cropped image was saved in PNG format. The oral and maxillofacial surgeons who cropped the image data were completely unaware of the osteoporotic status of each patient as this information was concealed from them according to the experimental design.

### CNN model architecture

In this study, the evaluation was performed using the standard CNN models, including a residual neural network (ResNet)^19^ and EfficientNet^20^. ResNet, invented by He et al.^19^, won the classification task of the ILSVRC2015 Challenge. Generally, deepening the network layer improves the accuracy of image identification, but conversely, a network layer that is too deep reduces the accuracy. To deal with this issue, we introduced a learning method called residual learning that involves a network that can be deepened to 152 layers. This representative of the ResNet architecture has 18, 50, and 152 layers.

EfficientNet is a CNN that was proposed as a state-of-the-art image classification method on ImageNet data in 2019. Although the number of parameters is smaller than that of the conventional CNN model, EfficientNet is a high-speed and relatively accurate CNN model that uses EfficientNet-b0, -b3, and 0b7 models. For efficient model building^30^, it is possible to fine-tune the weights of existing models as initial values for additional learning; therefore, all CNNs were used to transfer learning with fine-tuned pre-trained weights using the ImageNet database^31^. The process of deep learning analysis was implemented using the PyTorch deep learning framework and the Python programming language.

### Clinical covariates

Patients in the high risk group for osteoporosis are generally female, older, and with lower body mass indices (BMIs)^32^. There are many other patient factors, but age, gender, and BMI were selected as factors that can be easily identified by dentists. BMI is given by weight in kilograms divided by the square of height in meters. Patients’ weight and height were recorded at the time of BMD measurement. Table 3 shows the clinical and demographic characteristics of the patients in this study.

**Table 3:** Clinical and demographic characteristics of the patients.

	Osteoporosis	Non-osteoporosis	*P* value
	(T-score ≦ − 2.5)	(T-score > − 2.5)	
Number of patients	237	541	
**Sex**			
Female	223 (28.7%)	346 (44.5%)	< 0.0001
Male	14 (1.8%)	195 (25.1%)	
Mean age, years (SD)	76.9 (7.2)	68.5 (13.7)	< 0.0001
BMI, kg/m^2^ (SD)	21.2 (3.4)	22.5 (3.7)	< 0.0001

### Architecture of the ensemble model

**Figure 3:**
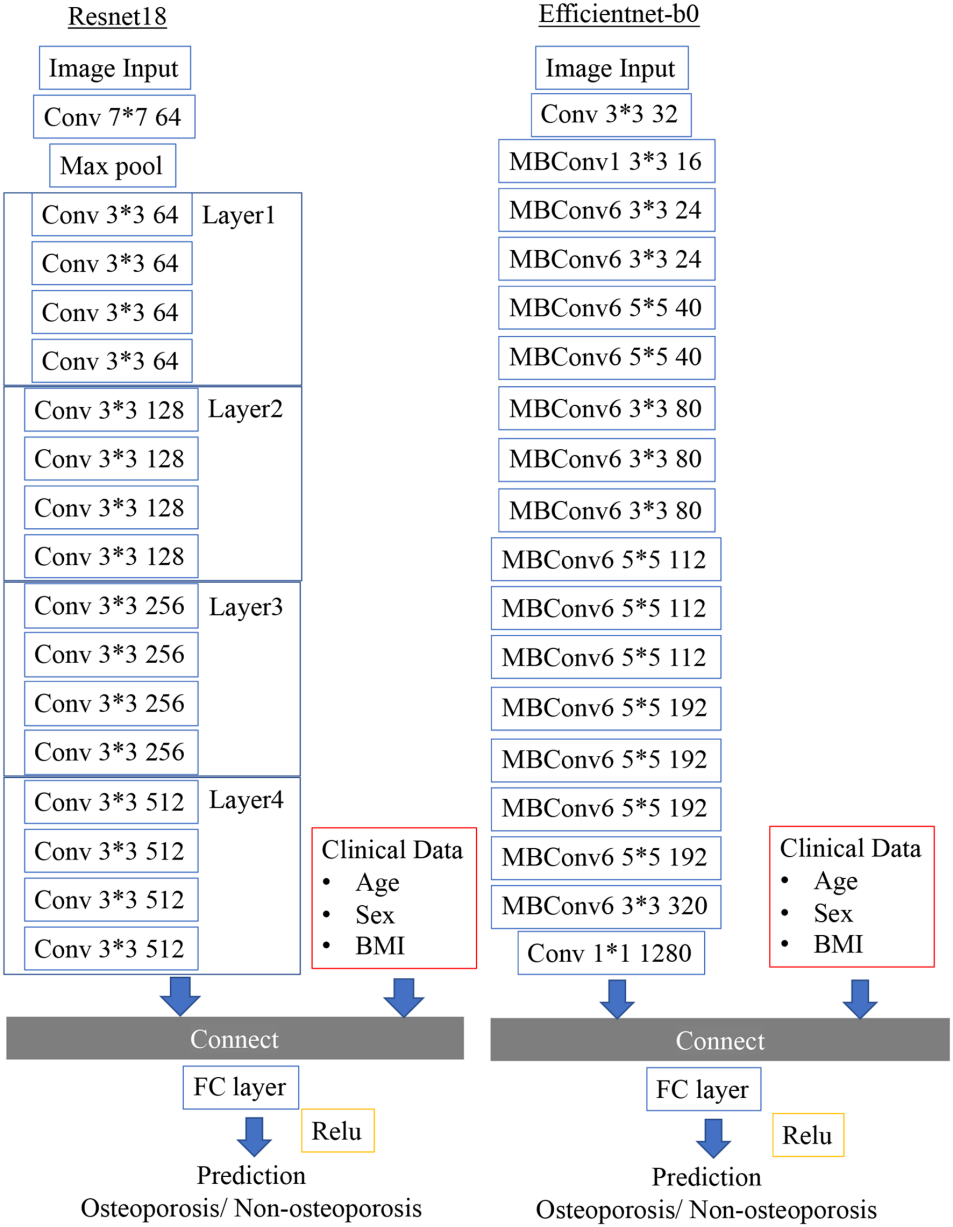
Neural network architecture that ensembles image data and clinical covariates. As representative models, ResNet18 and EfficientNet-B0 models are shown.

We also constructed an ensemble model that adds patient clinical factors to the deep learning analysis of X-ray images. In preparation, we preprocessed the structural data. Age and BMI were translated into mean normalization, and sex was translated into a one-hot vector representation. As a result, a 1 × 4 dimensional vector was created. Extracted from the convolutional layers in the CNN of the image, the one-dimensional reshaped result and the 1 × 4 dimensional data created from the structural data were combined. The image data processed by CNN and the combined data with clinical covariates were then passed as fully connected layers. The predictions of the final osteoporosis identification model were output using the rectified linear unit (ReLU) activation function (Fig. 3).

### Data augmentation

Various data augmentation techniques have been used to prevent overfitting owing to the small dataset size. During learning, data augmentation was applied only to the training image data when the images removed in batches. The training images were randomly rotated from − 25 to + 25, with a 50% chance to flip vertically and 50% chance to flip horizontally. The darkness was randomly changed from − 5 to + 5%, and the contrast was changed from − 5 to + 5%. Each training image was processed with a 50% chance of data augmentation.

### Model training

The model training was generalized using *k*-fold cross-validation. The images selected as the dataset were split using the stratified *k*-fold approach, which splits the training data, validation data, and testing data while maintaining the correct label percentages. The training algorithm used *k* = 5 for *k*-fold cross-validation to avoid overfitting and bias and to minimize generalization errors. The data were divided into five sets, and the testing data consisted of 156 images. In each fold, the data set was split into a separate training and validation sets at a ratio of 8:1. The selected validation data set was independent from the training set and was used to evaluate the training status. After completing one model training step, we performed similar validations five times with different testing data.

### Deep learning procedure

All deep learning models were trained and analyzed by using the 64-bit Ubuntu 16.04.5 LTS operating system on a workstation with 8 GB memory and an NVIDIA GeForce GTX 1080 8 GB graphics processing unit. The optimizer, weight decay, and momentum were common among all the CNNs. In this study, the optimizer used stochastic gradient descent, with a weight decay of 0 and momentum of 0.9. Learning rates of 0.001 and 0.01 were used for both ResNet and EfficientNet. All the models analyzed a maximum of 100 epochs. We used the early stopping method to terminate the data training to prevent overfitting if the validation error did not update 20 times in a row. This process was performed 30 times on all CNN models for statistical evaluation.

#### Performance metrics and statistical analysis

Our key performance indicators, namely, the osteoporosis discrimination accuracy, precision, recall, specificity and F1 score, are defined by Eqs. (1), (2), (3), (4), and (5), respectively, which account for the relations between the positive labels of the data and those given by the classifier. We also calculated the ROC curve and measured the AUC.

1 $${\text{accuracy}} = \frac{{{\text{TP }} + {\text{ TN}}}}{{{\text{TP }} + {\text{ FP }} + {\text{ TN }} + {\text{ FN}}}},$$

2 $${\text{precision}} = \frac{{{\text{TP}}}}{{{\text{TP }} + {\text{ FP}}}},$$

3 $${\text{recall}} = \frac{{{\text{TP}}}}{{{\text{TP }} + {\text{ FN}}}},$$

4 $${\text{specificity}} = \frac{{{\text{TN}}}}{{{\text{TN }} + {\text{ FP}}}},$$

5 $${\text{F}}1{\text{ score}} = 2 \times \frac{{{\text{precision }} \times {\text{ recall}}}}{{{\text{precision }} + {\text{ recall}}}}.$$

here TP and TN represent the numbers of true positive and true negative results, respectively, and FP and FN represent the numbers of false positives and false negatives, respectively.

$$Hedges^{\prime}g = \frac{{|M_{1} - M_{2} |}}{s},$$

$$s = \sqrt {\frac{{(n_{1} - 1)s_{1}^{2} + \left( {n_{2} - 1} \right)s_{2}^{2} }}{{n_{1} + n_{2} - 2}}} .$$

M_1 _and M_2_ are the means for the ensemble and image-only models; s_1 _and s_2_, respectively, are the standard deviations for the ensemble and image-only models; and n_1_ and n_2_, respectively, are the numbers for the ensemble and image-only models.

Statistical analyses were performed for each performance metric with the use of JMP Statistics Software Package Version 14.2.0 for Macintosh (SAS Institute Inc., Cary, NC, USA). *P* < 0.05 was considered statistically significant, and 95% confidence intervals were calculated. Parametric tests were performed based on the results of the Shapiro–Wilk test. The effect sizes were calculated as Hedges' g (unbiased Cohen's d). The effect size was determined as follows based on the criteria proposed by Cohen and expanded by Sawilowsky^33^: a very small effect was 0.01, small effect was 0.2, medium effect was 0.5, large effect was 0.8, very large effect was 1.0, and huge effect was 2.0.

#### Visualization of the computer-assisted diagnostic system

Gradient-weighted class activation mapping (Grad-CAM) is a technology that visualizes important pixels by weighting the gradient with respect to the predicted value^34^. It shows information that is significant for identification: the high gradient of the input to the last convolutional layer. Guided Grad-CAM is a combination of Grad-CAM and backpropagation visualization techniques that are useful for identifying detailed feature locations. The feature area visualization was reconstructed from the last convolution layer of each CNN in this study.

## Supplementary Information

Supplementary Information.
