# Supplementary material for: Identification of osteoporosis using ensemble deep learning model with panoramic radiographs and clinical covariates
Source: Sci Rep. 2022 Apr 12;12:6088. doi: 10.1038/s41598-022-10150-x (PMC9005660; doi:10.1038/s41598-022-10150-x)
Supplement: Supplementary file 1 — Supplementary Information. [file 41598_2022_10150_MOESM1_ESM.docx]

Identification of osteoporosis using ensemble deep learning model with panoramic radiographs and clinical covariates

Shintaro Sukegawa ^1,2,^ *, Ai Fujimura ^1^, Akira Taguchi ^3^, Norio Yamamoto ^4^, Akira Kitamura ^5^, Ryosuke Goto ^5^, Keisuke Nakano ^2^, Kiyofumi Takabatake ^2^, Hotaka Kawai ^2^, Hitoshi Nagatsuka ^2^, Yoshihiko Furuki ^1^

^1^Department of Oral and Maxillofacial Surgery, Kagawa Prefectural Central Hospital, 1-2-1, Asahi-machi, Takamatsu, Kagawa 760-8557, Japan

^2^Department of Oral Pathology and Medicine, Graduate School of Medicine, Dentistry and Pharmaceutical Sciences, Okayama University, Okayama 700-8558, Japan

^3^Department of Oral and Maxillofacial Radiology, School of Dentistry, Matsumoto Dental University, 1780 Hirooka Gobara, Shiojiri, Nagano, 399-0781, Japan

^4^Department of Epidemiology, Graduate School of Medicine, Dentistry and Pharmaceutical Sciences,
Okayama University, Okayama, 700-8558, Japan

^5^Search Space Inc., Tokyo 151-0072, Japan

**Appendix**

**Figure S1.** ROC curves for each of the EfficientNet and ResNet models with dental panoramic radiographs alone and the CNN analysis with dental panoramic radiographs combined with clinical covariates analysis.

**
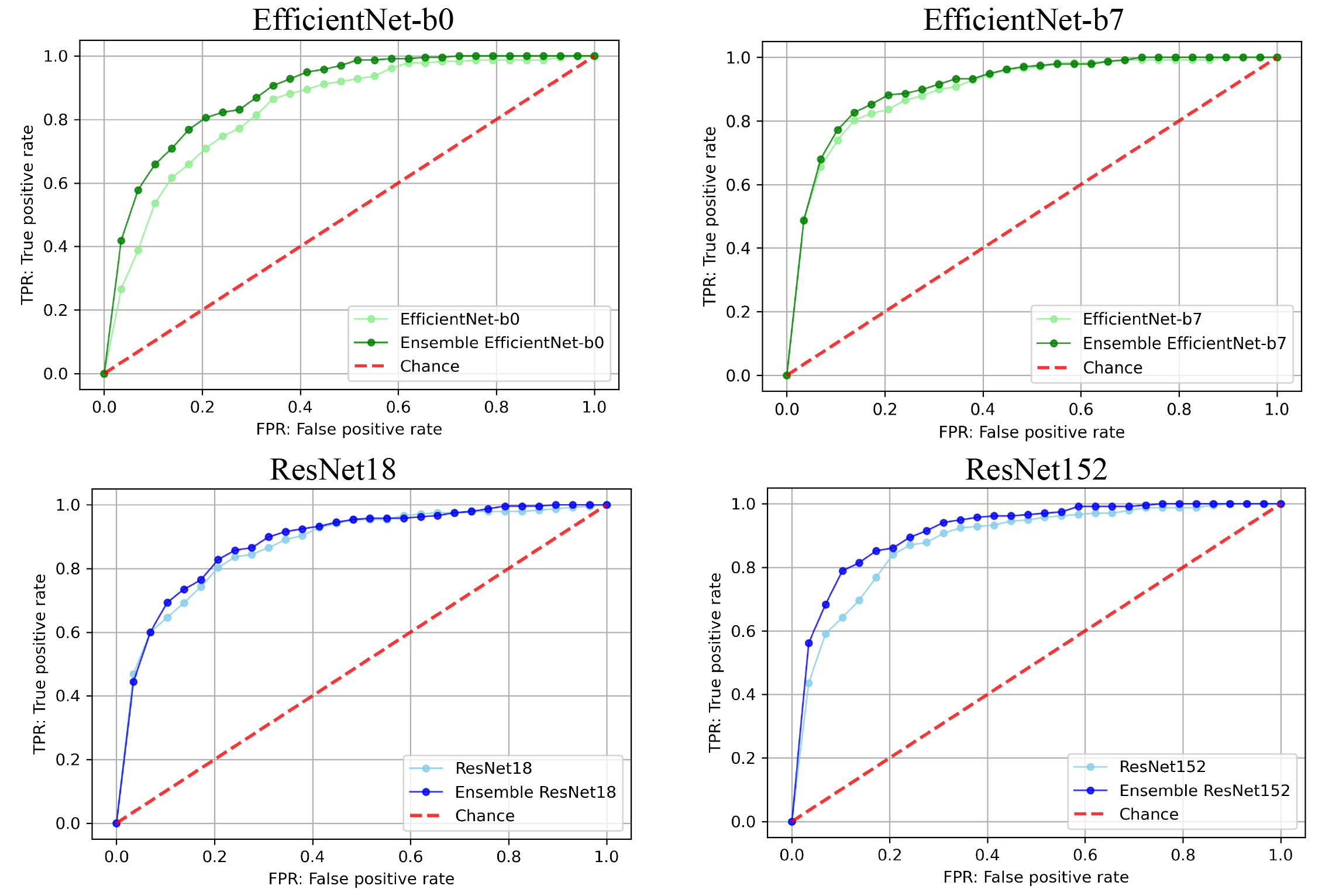
**
